# Review of recent advances in managing periocular skin malignancies

**DOI:** 10.3389/fonc.2024.1275930

**Published:** 2024-03-04

**Authors:** Daniel C. Trotier, Leslie Huang, Suzanne W. van Landingham, Adam R. Burr, Vincent T. Ma

**Affiliations:** ^1^ University of Wisconsin School of Medicine & Public Health, Madison, WI, United States; ^2^ Department of Medicine, Division of Hematology, Medical Oncology, and Palliative Care, University of Wisconsin-Madison, Madison, WI, United States; ^3^ Department of Ophthalmology and Visual Sciences, University of Wisconsin-Madison, Madison, WI, United States; ^4^ Department of Human Oncology, University of Wisconsin-Madison, Madison, WI, United States; ^5^ University of Wisconsin Carbone Cancer Center, University of Wisconsin-Madison, Madison, WI, United States; ^6^ Department of Dermatology, University of Wisconsin-Madison, Madison, WI, United States

**Keywords:** periocular malignancy, cutaneous squamous cell carcinoma, basal cell carcinoma, melanoma, Merkel cell carcinoma, sebaceous gland carcinoma, neoadjuvant therapy, periorbital malignancy

## Abstract

Management of cutaneous malignancies can be particularly challenging when they are located in the periocular region. The standard of care for localized disease is complete surgical excision, but this may not be possible without significant disruption to visual structures and facial appearance. Definitive radiation may be an option for some patients who cannot or do not wish to undergo surgery. Advances in systemic treatment options for locally advanced and metastatic skin cancers in the past 10 years have prompted investigation into neoadjuvant treatment of periocular cancers. The use of chemotherapy, immune checkpoint inhibitors, and targeted therapies have all been reported with varying degrees of success. For many patients, targeted therapies or immune checkpoint inhibitors should be considered depending on the cancer type, symptoms, and goals with the input of a multidisciplinary cancer care team. In this article, we systematically review the latest updates in surgical, radiotherapeutic, and medical management of periocular malignancies.

## Introduction

Skin cancers frequently occur in the periocular area due to chronic ultraviolet (UV) exposure on the head and neck. Basal cell carcinoma is the most common periocular skin cancer, followed by squamous cell carcinoma, malignant melanoma, Merkel cell carcinoma and sebaceous gland carcinoma (1). Cancers in the periocular region and the techniques used to treat them carry significant morbidity and quality of life implications (QOL) (2). Current guidelines for localized skin cancers recommend excision or definitive radiotherapy which is curative for most patients. Invasion of the orbit by periocular skin cancers can necessitate orbital exenteration which significantly affects vision and appearance (3, 4). Due to the morbidity and QOL impact with definitive treatment, periocular malignancies represent a unique and challenging clinical situation.

Until recently, there have been limited effective systemic therapies for these common skin cancers. Chemotherapy has had limited success in treating skin cancers and is not effective for basal cell carcinoma or malignant melanoma, and its current role is limited to select scenarios with squamous cell carcinoma, Merkel cell carcinoma, and sebaceous gland carcinoma. Advances in understanding of the molecular pathways driving cancer development and the role of the immune system in cancer surveillance has led to the rise of immunotherapy and targeted therapies for locally advanced and metastatic skin cancers. These developments have prompted investigation into the possible use of neoadjuvant systemic therapy to minimize the morbidity of definitive therapies and improve long-term outcomes. Recent clinical studies have begun to provide an evidence base for the use of neoadjuvant targeted and immunotherapies for skin cancers and may soon result in changes to treatment guidelines of these malignancies. In this review article, we briefly review surgical and radiotherapeutic approaches which constitute the current standard of care treatments for periocular malignancy and review the role for systemic therapy for tumors not amenable to curative intent surgery or radiation.

## Surgical management of periocular malignancies

The goal of surgical treatment for periocular malignancies is complete removal of the tumor while minimizing recurrence, metastasis, morbidity, and mortality. The challenge lies in achieving both complete eradication of the tumor and optimal functional and cosmetic outcomes for the patient. The periocular region presents unique challenges to the treating physicians, given the close proximity of important anatomical structures. Preservation of the globe, where possible, is paramount.

Complete surgical excision is the mainstay of treatment for localized tumors. Excisional biopsy with pre-determined margins is appropriate for suspected non-melanoma skin cancer on some parts of the body. Reconstruction with tissue rearrangement is delayed until pathological margins are confirmed as negative. For example, meta-analysis has shown that excision of small (<2 cm diameter), non-morpheaform basal cell carcinoma with 3-mm surgical margins yields a 95% cure rate (5). This technique is rarely used near the eye, however, since it may result in a larger soft tissue defect than other techniques, and preservation of as much healthy tissue as possible helps ensure successful reconstruction. Furthermore, the risks and stakes of recurrence are relatively high (6).

Surgical excision with margin control through microscopic frozen-section control may also be used. The frozen section technique is used to identify the extent of the lesion by excising full-thickness sections along the clinically apparent tumor, along with an additional 1-2 mm of tissue beyond the margins of the lesion (7). Pathologic examination of the excised specimen allows for the evaluation of residual tumor. Surgical resection is then continued until the margins show no histological evidence of tumor remaining.

While use of conventional histopathological examination with “bread-loaf” style sections can be used for low-risk tumors (such as nodular basal cell carcinoma) and may be easier to coordinate in some centers, Mohs micrographic surgery and other forms of peripheral and deep en face margin assessment (PDEMA) are preferred. National Comprehensive Cancer Network (NCCN) clinical guidelines support use of these techniques for periocular basal cell carcinoma and squamous cell carcinoma, as location in the periocular region categorizes them as high risk (8). The advantage of the Mohs technique and other PDEMA techniques is that they allow histopathological inspection of the specimen’s complete margin via removal of tissue in thin layers (9, 10). Recurrence rates for eyelid basal cell carcinomas after Mohs surgery have been reported as low as 0.6%, which demonstrates the reliability of micrographic surgery while also sparing maximal adjacent normal tissue (9).

Complete description of surgical management algorithms and suggested surgical margins of cutaneous malignancies is outside of the scope of this review and will vary based on tumor histology and individual patient factors (**Table 1**). In brief, national guidelines for basal cell and squamous cell carcinoma support complete excision using Mohs surgery or other forms of PDEMA, followed by surgical reconstruction (3, 4). Sebaceous cell carcinoma is managed similarly, though map biopsies of the conjunctiva may be indicated to evaluate for pagetoid spread and a sentinel lymph node biopsy (SLNB) to evaluate for nodal metastasis may be indicated for tumors >10 mm in width or that are recurrent (11). Melanoma management is correlated with Breslow thickness and involves complete excision using permanent sections for margin control via a “slow Mohs” technique with use of permanent fixation for evaluation of margins or staged excision, and consideration of SLNB for depth >1 mm. Detailed surgical margin recommendations for melanoma are based on Breslow thickness and outlined in NCCN guidelines. Merkel cell carcinoma is typically managed with SLNB and complete excision with Mohs surgery or wide local excision with 1-2 cm margins (12, 13).

**Table 1:** Favored surgical techniques for surgical management of periocular cutaneous malignancies.

Favored surgical techniques	Basal cell carcinoma	Squamous cell carcinoma	Sebaceous cell carcinoma	Melanoma	Merkel cell carcinoma
**Pre-operative map biopsy**	–	–	Yes	–	–
**Wide local excision**	–	–	–	–	Yes (1-2cm margin)
**Excision with frozen section margin control**	Yes (especially for nodular varieties)	–	–	–	–
**Staged excision with permanent fixation for margin control**	–	–	–	Yes	–
**Mohs surgery or other PDEMA techniques**	Yes	Yes	Yes	Yes (needs “slow Mohs” technique with use of permanent fixation)	Yes
**Sentinel lymph node biopsy**	–	–	Yes (tumor diameter >10 mm or recurrence)	Yes (Breslow thickness >1.0 mm)	Yes

Note that choice of surgical technique depends on tumor and patient factors, and more detailed algorithms may be found via the National Comprehensive Cancer Network (NCCN) website at www.nccn.org. Patients should be counseled on risks of progression or toxicity prior to planned surgery. Complex cases should be considered with a multidisciplinary approach of oculofacial surgeons, radiation and medical oncologists in accordance with patient’s preferences and goals.

PDEMA, peripheral and deep en face margin assessment

### Surgical and reconstructive considerations

**Figure 1:**
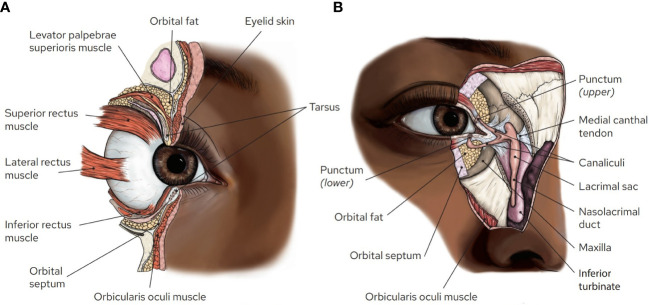
Orbital and ocular adnexal anatomy. **(A)** Sagittal section of the orbit illustrating the proximity of multiple structures within the eyelids and anterior orbit. Note the orbital septum, which separates the preseptal tissues from the orbital tissues, and the presence of fat that cushions the orbital contents prevents establishing clear margins within the orbit. **(B)** *En face* view of the medial orbit with portions of the skin, muscle, orbital septum, medial canthal tendon, and bone cut away to illustrate the lacrimal drainage system. Artwork by Rae Senarighi.

Reconstruction following surgical excision of a periocular cutaneous malignancy is a crucial component of restoring optimal function and the appearance of the eye. Surgical resection and reconstruction of eyelid malignancies is particularly challenging because of the proximity to vital anatomical structures (**Figure 1**). Understanding what structures may be involved by a malignancy can help the surgeon create a reconstructive plan and determine which tumors are less amenable to surgery and may benefit from adjuvant or neoadjuvant treatments. This section will review important periocular anatomical structures and discuss findings that may contraindicate conventional surgical management.

The eyelids have complex anatomy (**Figure 1**). Reconstruction of the lids strives to maintain the ability of the lids to blink, limit lagophthalmos (inability to close the eye), maintain an unobstructed visual axis, and maintain an appropriate tear film. Proper blinking and tear film is necessary to keep the surface of the eye comfortable and the vision clear. These functions require an intact orbicularis oculi muscle, adequate height of the upper and lower lids, and functional lacrimal excretory and drainage systems. Poor blink and lagophthalmos can lead to dry eye and exposure keratopathy, which can cause corneal scarring and vision loss in severe cases. The eyelashes must be directed away from the eyes to prevent discomfort and corneal damage. Numerous techniques are employed by the oculofacial surgeon to reconstruct the lids following Mohs or other excisional surgery. Full- or partial-thickness defects of the lids can be managed by various techniques which may include primary layered closure, full-thickness skin grafting, myocutaneous flaps, and vascular pedicle flaps (such as paramedian forehead flaps).

Complete loss of the lower eyelid presents some challenges but can be readily managed by techniques that make use of the larger upper eyelid, for example the eyelid-sharing 2-staged Hughes flap. A much more challenging defect would be a complete loss of the upper eyelid. Because the upper lid is taller than the lower lid, eyelid-sharing procedures offer less adequate coverage and the surgeon may need to raise more elaborate flaps (14). Post-operative dry eye, ptosis, and other problematic ophthalmic sequelae are much more likely with complete upper lid reconstruction.

An important anatomical structure that requires special considerations for resection and reconstruction is the lacrimal drainage system (**Figure 1**). Tears leaving the surface of the eye enter the lacrimal drainage system via the lacrimal punctum, located along the medial eyelid margin, and then flow through the lacrimal canaliculi, which is fairly superficial within the medial eyelid margin. They then enter the lacrimal sac, which is deep to the inferior portion of the medial canthus, through the nasolacrimal duct, and into the nose. Tumors involving the medial canthal region may necessitate partial or complete removal of the lacrimal drainage system. Primary reconstruction of the lacrimal system can often be achieved with silicone nasolacrimal intubation if a small portion is disrupted (15). If too much of the lacrimal system is excised to allow for primary reconstruction, the patient typically undergoes cutaneous reconstruction without repair of the lacrimal system and monitored for the development of symptomatic epiphora. For some elderly patients who suffer from age-related chronic dry eye, epiphora may be less bothersome; however, patients must be counseled on the possibility of chronic tearing (16). In the case that the patient develops symptomatic epiphora, a conjunctivo-dacryocystorhinostomy (CDCR) with Jones tube placement can be considered, essentially using a glass tube to replace the native canalicular system (15). Many surgeons delay placement of these tubes for at least a year following excision of a malignancy due to concerns that premature placement may spread residual tumor cells into the nasal cavity. Given the challenges of lacrimal system reconstruction and burden of chronic epiphora, involvement of the lacrimal system is sometimes cited as an indication for use of neoadjuvant therapies.

**Figure 2:**
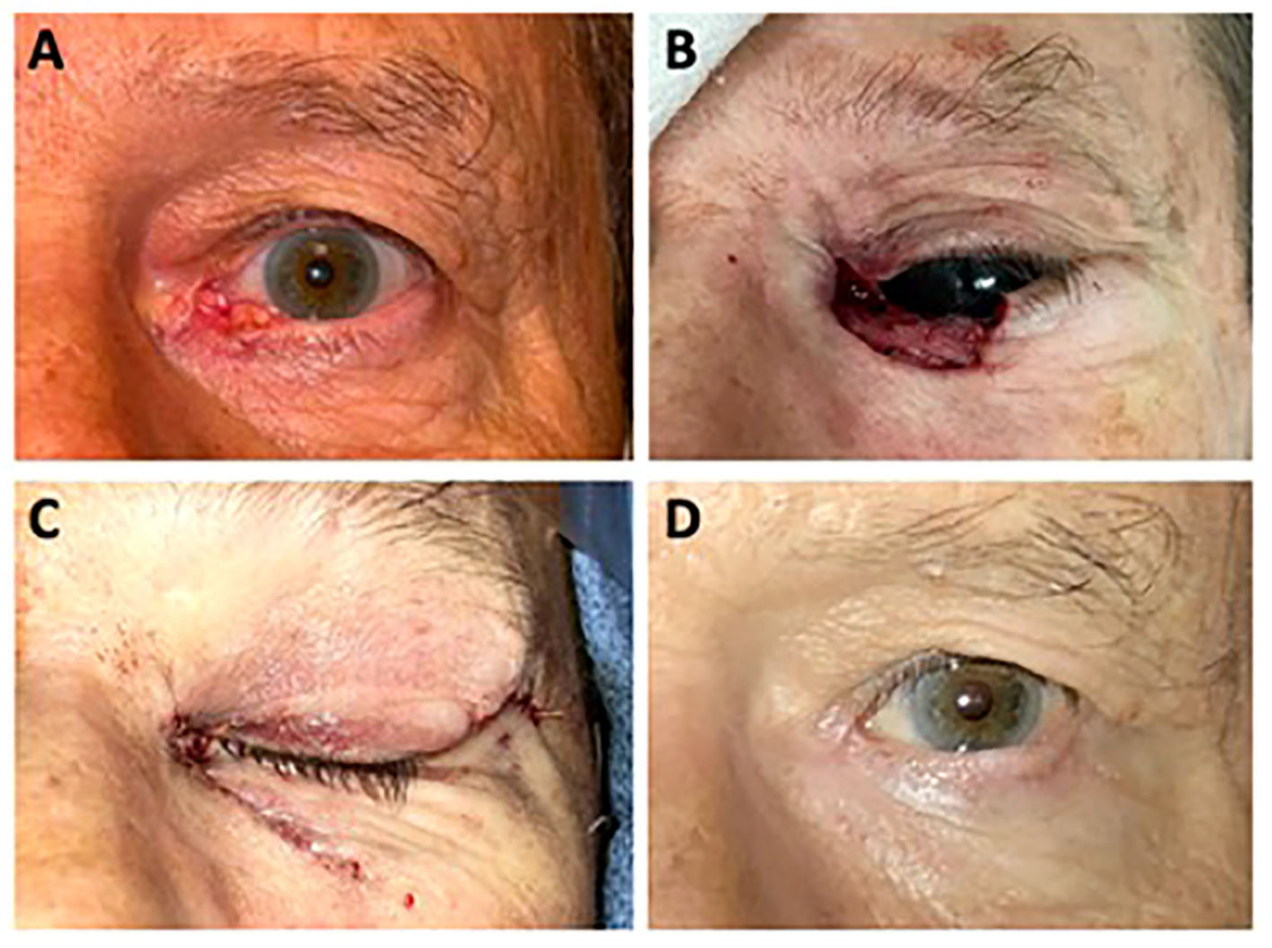
**(A)** A 69-year-old man presented with a non-healing, ulcerated left lower lid lesion with raised, pearly, telangiectatic borders. Biopsy revealed basal cell carcinoma. **(B)** Surgical defect of Mohs micrographic surgery. **(C)** Immediately following surgical reconstruction of the left lower lid margin using a semicircular Tenzel flap and reconstruction of the left upper lid margin with a medial tarsal strip. **(D)** 12 months following Mohs surgery and reconstruction.

A case illustrating lacrimal system involvement can be found in (**Figure 2**). This is a 69-year-old male who presented with two years of ocular irritation attributed to a non-healing abrasion. Examination revealed an ulcerated lesion with raised, pearly, telangiectatic borders measuring 1.2 cm x 0.6 cm. Clinically, it appeared to involve the medial canthus and medial lower eyelid with complete effacement of the lower punctum and canaliculus. Biopsy revealed basal cell carcinoma. He underwent Mohs surgery, which ended in clear margins and yielded a defect measuring 3.0 cm x 1.5 cm. The defect involved a large portion of the medial canthus and medial upper and lower eyelids, including the complete upper and lower lacrimal canaliculus. Because of the extent of lacrimal system involvement, primary reconstruction of the lacrimal system was not feasible. The upper eyelid was reconstructed with creation of a medial tarsal strip and resuspension to the periosteum of the medial orbital rim, and the lower eyelid was reconstructed using a semicircular Tenzel flap and resuspension to the periosteum of the medial canthus. While he noted epiphora at his 1-year follow-up visit, he was satisfied with his result and declined CDCR surgery.

Another important structure to consider when managing periocular malignancies is the orbital septum (**Figure 1**). This is a fibrous sheet that originates at the periosteum of the orbital rim and inserts into the tarsus and the lateral and medial canthal tendons. This layer forms a barrier between the orbital contents and the anterior or “preseptal” soft tissues. This boundary is important in the treatment of infection (e.g. preseptal vs. orbital cellulitis) and malignancies, as involvement of the orbital contents puts the eye at much greater risk. When a cutaneous malignancy has breached the septum, even by a small amount, achieving clear surgical margins becomes challenging to impossible. The orbital contents (eye, extraocular muscles, optic nerve, etc.) are surrounded and cushioned by soft, mobile, fat, which is less amenable to identifying histopathological margins than, for example, skin or muscle, which have a relatively fixed position.

**Figure 3:**
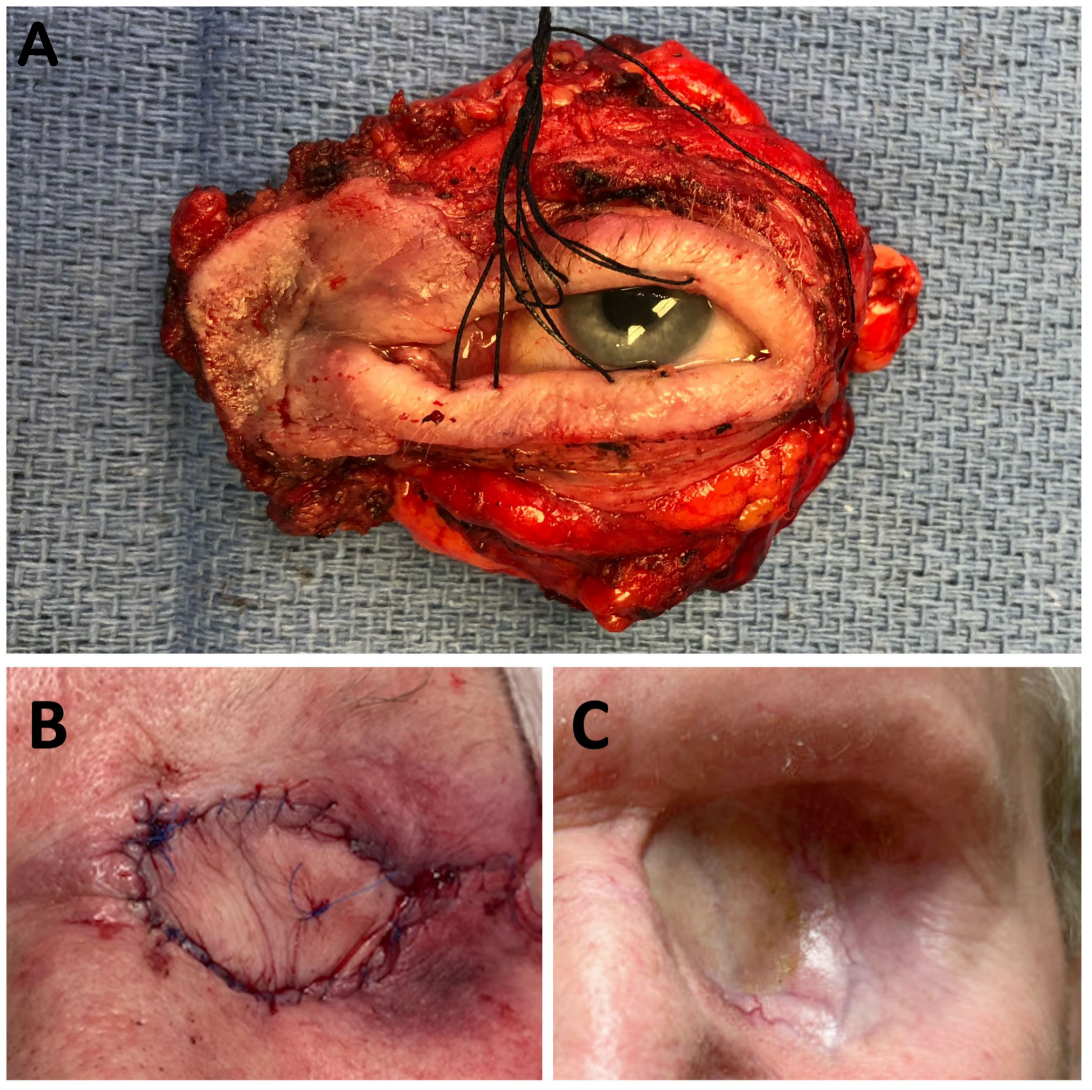
**(A)** Intra-operative exenteration specimen. The indication for exenteration was squamous cell carcinoma invasive to the orbit. **(B)** Immediate postoperative view of a 65-year-old male patient following orbital exenteration surgery for aggressive squamous cell carcinoma of the left lacrimal sac. The patient was reconstructed with a radial forearm free flap due to the need for post-operative adjuvant radiation. **(C)** Eight months after orbital exenteration.

Traditionally, violation of a tumor into orbital septum has been an indication for orbital exenteration (17). Exenteration involves removal of the entire contents of the orbit including the globe, extraocular muscles, orbital fat, and sometimes the eyelids (18). Reconstruction following exenteration can be achieved through spontaneous healing by granulation of the bony socket, with the use of a split-thickness skin graft, synthetic materials, or flaps (19). Healing by secondary intent can take up to a year. (**Figure 3**) demonstrates an intraoperative photo of an exenteration specimen from a 65-year-old patient with squamous cell carcinoma invasive to the orbit. Because adjuvant radiation therapy was planned, a vascular flap (radial forearm free flap) was used in reconstruction.

Aside from the obvious consequence of monocular vision loss, exenteration is disfiguring and can lead to subsequent psychological distress for the patient. A prosthesis can be made to mask the deformity, but as the eye does not move or blink, the naturalism of these devices is limited (20). For this reason, violation of the orbital septum or invasion of the orbit is commonly cited as a reason for using neoadjuvant therapy.

Finally, some periocular tumors, especially large or neglected tumors, can involve the facial bones. For example, deep medial canthal tumors can invade the sinuses via the lacrimal sac fossa or ethmoid bone, and larger, neglected basal cell carcinomas can invade the frontal bone and frontal sinus. Surgical resection of these tumors, which would necessitate resection of bone and sometimes large free flaps for reconstruction, may be disfiguring and achieving clear margins may be challenging to impossible. Involvement of bone is often a reason for use of adjuvant or neoadjuvant therapies.

In sum, the standard of care for periocular cutaneous malignancies is surgical resection with histopathological margin control, preferably using a PDEMA technique such as Mohs surgery. Very large tumors, tumors that invade the orbit, and tumors that involve important periocular structures such as the lacrimal system are less amenable to surgical resection, as surgical resection may result in unacceptable morbidity. Patient factors, such as suitability for general anesthesia and comorbidities affecting wound healing may also contraindicate surgical management. These more extensive malignancies may benefit from other treatment modalities, used in a definitive, adjuvant, or neoadjuvant manner.

## Radiation therapy for periocular malignancies

Radiation therapy has been used as either a definitive or adjuvant therapy for periocular malignancies for many decades. A wide range of radiation therapy techniques have been utilized for periocular malignancies including orthovoltage radiation, intensity modulated radiation therapy (IMRT), 3D-conformal radiation therapy, brachytherapy, protons, and electrons depending on the location of the lesion and modalities available at a given center (**Table 2**). The challenge of employing these techniques is to achieve adequate dosimetric coverage of the target while sparing the substructures of the eye appropriately, requiring large dose gradients precise treatment set-up.

**Table 2:** Favored radiotherapy techniques for management of periocular cutaneous malignancies.

Favored radiation techniques	Basal cell carcinoma	Squamous cell carcinoma	Sebaceous cell carcinoma	Melanoma*	Merkel cell carcinoma
**Electrons with eye shield**	Yes	Yes	Yes	–	Yes
**IMRT**	Yes	Yes	Yes	–	Yes
**IMPT**	Yes	Yes	Yes	–	Yes
**3DCRT**	–	–	–	–	–
**ENI**	–	Consider for T3/T4	–	–	Consider if no SLNB or if cN0 and T3/4 or LVSI

Surgical resection remains the standard for all tumor types. Radiation can be considered for tumors that are unresectable or to spare the globe or other morbidity.

*As a generally radioresistant tumor, radiation should not be routinely considered off clinical trial in the definitive treatment of melanoma.

IMRT, intensity modulated radiation therapy; IMPT, intensity modulated proton therapy; 3DCRT, 3-dimensional conformal radiotherapy; ENI, elective nodal irradiation; SLNB, sentinel lymph node biopsy; LVSI, lymphovascular space invasion.

Periocular cutaneous malignancy is predominantly treated surgically as above. However, definitive radiation therapy may be considered due to patient preference, lack of eligibility for surgery, or high anticipated functional morbidity of surgery. While there are no randomized trials comparing surgery to radiation therapy in periocular tumors, the relative control rates can be inferred from a recent meta-analysis in cutaneous non-melanomatous skin cancer showing a recurrence rate of 1.8% for radiation versus 0.2% for Mohs surgery (21). A single randomized trial from the 1980s including basal cell carcinoma patients showed a recurrence rate of 0.7% for surgery versus 7.5% for radiation, providing an upper bound in the differences between modalities since modern radiation series have shown lower rates of recurrence (22). In peri-orbital sites, a retrospective study of 128 basal cell carcinomas of the eyelids and canthi showed a 100% local control rate using superficial X-rays (23). A recent retrospective review of 42 patients (30 definitive, 12 adjuvant) with squamous cell carcinoma of the eyelid treated with superficial X-rays or electrons with appropriate eye shields showed no difference in local control between patients receiving primary (89%) or adjuvant radiation therapy (86%) with no grade 3 complications in either arm. High rates of local control have also been achieved in sebaceous gland carcinoma of the eyelid without grade 3 toxicity (24). Thus, definitive radiation can be effectively employed for appropriately selected periocular malignancies; however, careful patient selection is needed.

Radiation therapy has also been utilized in the adjuvant setting to decrease the necessity for orbital exenteration in more advanced periocular tumors. The strongest indication for adjuvant radiation therapy is a positive margin, given the high recurrence risk. After a negative margin resection, recommendations for adjuvant therapy include a multi-disciplinary discussion with attention to close margins, perineural invasion, adenoid cystic histology, deep invasion, poorly differentiated histology, or bone invasion. A retrospective study of 20 patients treated by globe-sparing surgery followed by proton radiation showed a 100% local control rate when surgery was followed by a median of 60 Gy relative biologic effectiveness (RBE) (25). In 13 patients, they utilized a corneal deviation technique by diverting their gaze away from the location of the radiation to limit the dose to the cornea. In this study, they found grade 3 epiphora in 3 patients, grade 3 exposure keratopathy in 3 patients, and four patients had decreased visual acuity. They found patients with higher doses to the cornea were at higher risk of late toxicity and recommended limiting the dose to the cornea to 35 Gy RBE. Similar eye sparing approaches using particle therapy have also been reported, including for adenoid cystic carcinoma with good local control (26, 27). Thus, with appropriate dose constraints, radiation therapy can be utilized to preserve vision and decrease recurrence.

With the tools of modern radiation therapy, many periocular lesions can be addressed while preserving the function of the eye. To define which cases can undergo a more limited surgery followed by adjuvant radiation therapy an understanding of the available tools of radiotherapy and their limitations is necessary. To simplify this topic somewhat for discussion, radiation techniques can be separated into techniques for superficial tumors such as electronic brachytherapy, and orthovoltage X-ray therapy and those for deeper tumors such as IMRT and proton therapy. Superficial techniques are better suited to treating the eyelid but will not be able to treat beyond 2.5 cm deep into the orbit for perineural invasion (PNI) or more invasive lesions. Conversely, IMRT and intensity modulated proton therapy (IMPT) will struggle to spare the globe while treating the lid due to the inability to physically shield photons or protons (**Table 3**).

**Table 3:** Clinical considerations affecting choice of radiotherapy technique for periocular malignancies.

Favored radiation techniques	Eyelid lesion	Superficial lesion	Deep lesion (>2.5 cm)	PNI	Nodal regions
**Electrons with eye shield**	Yes, preferred	Yes	–	–	–
**IMRT**	Possibly with acrylic shield	Yes	Yes	Yes	Yes
**IMPT**	–	Yes	Yes	Yes	Yes
**3DCRT**	–	–	–	–	–

IMRT, intensity modulated radiation therapy; IMPT, intensity modulated proton therapy; 3DCRT, 3-dimensional conformal radiotherapy; PNI, perineural invasion.

As many cutaneous neoplasms involve the lid without deep invasion, the use of orthovoltage X-rays or electrons can be an ideal solution. A comparison of these techniques is discussed elsewhere; however, we will focus on electrons, as they are much more widely available (28). Both techniques utilize shielding to be most effective. While sometimes an over eye, wax coated lead shield will work, often an internal eye shield will be required to shield the eye and simultaneously reach treatment doses. Electron beam energies of 6 and 9 MeV can be effectively shielded with a 3 mm Tungsten eye shield. Given the need for 0.5 cm of bolus to achieve full dose at the surface, one can treat to 2.5 cm of depth with this approach. The Tungsten eye shield is coated in aluminum to decrease excess backscatter onto the lid, while the acrylic coating is used for comfort. Tungsten shields of 3 mm thickness with 0.5 mm aluminum prevented 97% of beam transmission (29). The eye shield is easily placed in most patients by anesthetizing the eye with a topical anesthetic (e.g. proparacaine 0.5%) and then gently deflecting the eyelids to place the shield, similar to a contact lens (30). Two disadvantages of electron therapy are the need to prescribe to the 80-90% isodose line, creating a hotspot and the need for lateral margin for dose build-up, even in the setting of surface collimation. To ameliorate these issues and decrease scarring, we have utilized a mixed photon and electron plan.

**Figure 4:**
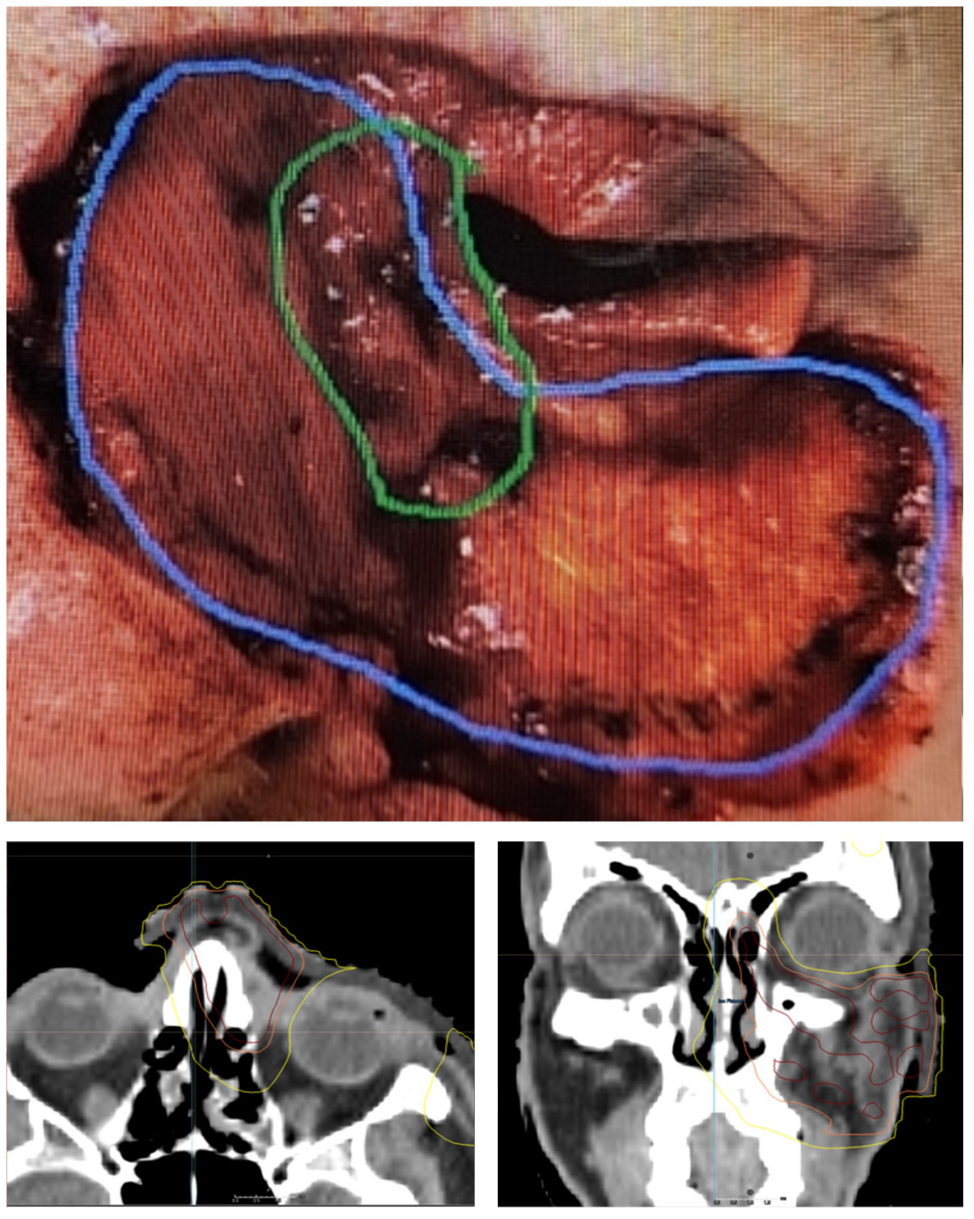
Photograph after Mohs resection of basal cell carcinoma with densely positive margin in the area of green and scattered cells on the margin in the blue area. Radiation plan delivering 50 Gy in 20 fractions to the resection bed with 55 Gy in 20 fractions to the area of positive margin. Yellow isodose line is 32 Gy, Orange isodose line is 95% isodose line of 50 Gy and 95% red is 95% isodose line of 55 Gy.

There are situations where treatment is needed to preserve the eye but shielding is not possible due to depth of tumor extension along the bony orbit or in the case where the eye remains sutured closed for healing. In these cases, it may be possible to achieve the desired dose to the area of positive margin while sparing the sensitive structures of the orbit using IMRT or IMPT (**Table 3**). An example of the areas of concerning margin and associated radiation isodose lines are depicted in (**Figure 4**). To safely use these techniques a knowledge of the safe dose to the structures of the eye is needed. While there are dozens of reported toxicities and constraints, the European Particle Therapy Network (EPTN) has published consensus constraints on several organs at risk that can be used as a starting point and are summarized here (31). The constraints below assume standard fractionation and should be scaled if hypofractionation is used. Lacrimal gland toxicity can result in xeropthalmia and dry eye syndrome. To avoid this risk, a mean of 25 Gy was suggested by EPTN though constraints vary across other studies (32). The cornea is a critical organ at risk for optical function across several studies, with toxicity to this organ at risk often resulting in vision changes or vision loss. The EPTN consensus is to limit dose to D_0.03cc_ < 50 Gy; however, we have generally adopted the more conservative D_0.03cc_ < 35 Gy constraint from MD Anderson Cancer Center, which has been achievable with corneal deviation technique (25). The EPTN makes no formal recommendation for conjunctival constraint; however, we will often use a D_0.03cc_ of 42 Gy. A retinal constraint of D_0.03cc_ < 45 Gy and an optic nerve constraint of D_0.03cc_ < 55 Gy were recommended. The recommended lens constraint is D_0.03cc_ < 45 Gy; however, this is not usually achievable without creating substantial horns of dose and pushing dose into other organs at risk. As cataract surgery is well tolerated, we generally omit this constraint. A simpler and more conservative method used for years at our institution is to split the globe into an anterior and posterior half with a conservative constraint of 30 Gy to the anterior globe and 45 Gy to the posterior. An understanding of the feasibility of achieving these constraints requires experience but is crucial to being able to decide on an upfront eye preserving strategy.

One important topic in the treatment of periocular malignancies is dose and fractionation. The doses required for the definitive and adjuvant radiation therapy for cutaneous squamous and basal cell carcinoma can be taken from the American Society for Radiation Oncology (ASTRO) consensus guidelines for a variety of fractionations (30). The dose chosen should reflect the anticipated microscopic risk and size of the lesion. In the periocular region, definitive doses can range from 60-70 Gy in 2 Gy fractions with 60 Gy generally very adequate for a small basal cell carcinoma and 66-70 Gy potentially necessary for larger squamous cell carcinomas. Adjuvant doses can range from 50-66 Gy using conventional fractionation, again depending on the risk and histology. Late effects are proportional to fraction size and total dose, so when possible more fractionated regimens are likely to lead to better cosmetic and functional outcomes. Hypofractionated courses are sometimes chosen due to travel distance, patient age, or comorbidity. Both 51 Gy in 17 fractions and 50-55 Gy in 20 fractions are well tolerated and maintain a biologically effective dose (BED) close to 100 Gy, which has been associated with an 80% chance of good cosmetic outcome in a more generalized non population (33). Overall, the preference should be for conventional fractionation in this sensitive location but the realities of advanced cutaneous malignancy mean that this will not always be feasible and systemic treatment should be considered.

There are select instances where radiotherapy may be administered concurrently with chemotherapy, namely for SCC. Definitive radiotherapy given with platinum-based chemotherapy (cisplatin or carboplatin) has been studied in unresectable cutaneous SCC of the head and neck. Doses of 70 Gy were administered with either weekly cisplatin or carboplatin and yielded a high complete response (CR) rate of 63% (34). Concurrent chemoradiotherapy may also be utilized for SCC with nodal involvement in patients who are not surgical candidates, or in the adjuvant setting in patients with residual disease or extracapsular extension detected following attempted resection. Radiotherapy has also been combined with EGFR inhibitors such as cetuximab, with or without additional chemotherapy for unresectable SCC or following surgery (35).

In summary, radiation therapy remains an important tool for the treatment of periocular malignancies that may grow in years to come, as it integrates more with rise of systemic neoadjuvant therapies. The availability of technology such as IMRT is ubiquitous and the availability of IMPT is increasing rapidly. More prospective studies will be crucial in optimally deploying radiation therapy with the goal of curing more periocular malignancies while sparing more patients from exenteration.

## Systemic therapies for periocular malignancies

### Basal cell carcinoma

Basal cell carcinoma (BCC) is the most common skin cancer, with a high proportion found on the sun-exposed face, head and neck (36–38). Risk factors include chronic UV exposure, fair skin and hair, freckles, and family history of BCC. The vast majority of cases are detected in early stages and can be managed with local excision or topical treatments such as imiquimod and fluorouracil. Topical treatments may not be appropriate depending on tumor size and proximity to the eyelids as they can cause corneal toxicity. Rarely, BCC can metastasize, but often can become deeply invasive with destruction of local structures. Early molecular studies identified that >90% of BCC’s bear mutations in the hedgehog signaling pathway making it a promising target for drug development (39). Genetic sequencing advances also identified BCC as the most highly mutated cancer due to mutagenesis from chronic UV exposure, with a median of 47.3 mutations/Mb (40). Initial immune checkpoint inhibitor trials identifying an association between high tumor mutational burden (TMB) and response rate have highlighted the use of immunotherapy for treatment of BCC (41).

### Chemotherapy for basal cell carcinoma

Prior to the development of novel agents, chemotherapy was the mainstay of treatment for BCC which had failed attempts with local therapy. Most early regimens contained platinum-based chemotherapy. Regimens with reported success include cisplatin or carboplatin with paclitaxel, etoposide, cyclophosphamide, vinblastine, 5-flurouracil, bleomycin, or methotrexate (42). A small case series reported 12 patients treated with cisplatin and/or doxorubicin was and reported 75% overall response rate with 33% complete responses (43). Chemotherapy is no longer a standard of care treatment for advanced basal cell carcinoma, and is only considered in patients who are not eligible for or have failed newer treatments (3).

### Hedgehog inhibitors for basal cell carcinoma

Hedgehog inhibitors (HHI’s) were first studied in locally advanced and metastatic BCC (laBCC and mBCC). The phase 1 STEVIE trial established the safety of vismodegib and found encouraging objective response rates (ORR) of 68.5% in laBCC and 36.9% in mBCC for an overall ORR of 58% (44). In the phase 2 ERIVANCE trial, treatment with vismodegib in 63 patients with laBCC and 33 patients with mBCC showed independently assessed ORR of 43% and 30%, respectively (45). The majority of patients with laBCC had tumor shrinkage but were not confirmed to have a response due to either missing data or subsequent progression. The phase 2 BOLT study of sonidegib, an alternative small-molecule HHI, in locally advanced and metastatic BCC have similar promising results, with ORR of 56% in laBCC and 8% in mBCC at a dose of 200 mg and an ORR of 46.1% and 17%, respectively, at a dose of 800 mg daily (46). The high response rate and tumor shrinkage seen in these trials prompted investigation into the possibility of neoadjuvant treatment of laBCC along sensitive anatomic locations, such as the face and periocular region, prior to curative intent surgery.

The phase 2 VISMONEO trial enrolled 55 patients with facial BCC that was deemed inoperable or operable with major functional or aesthetic risk. Subjects received vismodegib 150 mg daily for 4 to 10 months prior to planned surgical resection. Primary endpoint of this study was surgical downstaging, using a novel 6-stage scale ranging inoperable disease to complete response. Patients were treated for a median duration of 6.0 months. 44/55 (80%) of patients had surgical downstaging, with a mean tumor size reduction of 66%, and a 70.9% ORR by RECIST v1.1 (47). There was a significant proportion of complete responses (CR), with 27/55 patients (49%) exhibiting a clinical CR. No patients progressed while on vismodegib. Significant quality of life improvement was reported, with clinically meaningful improvement in quality of life assessment metrics. At 3-year follow-up, there was a significant relapse rate of 36.4% in the cohort of 44 patients who had responded to treatment with vismodegib. This may be due in part to the significant portion of patients who did not ultimately undergo surgery. Of the 27 patients with a complete clinical response, only 6 opted for scar excision (48). Overall, the high response rate and improvement in surgical staging provided proof of concept that neoadjuvant vismodegib for laBCC of the face may be a reasonable treatment approach for some patients.

The VISORB trial sought to specifically examine if neoadjuvant vismodegib in periocular BCC would help preserve visual organs and function. In this single center prospective trial, 34 patients with locally advanced periocular BCC were enrolled for whom surgical excision would require either orbital exenteration or loss of lacrimal drain function and loss of extraocular motility. The primary endpoint in this study was visual function measured using a novel Visual Assessment Weighted Score (VAWS), which was developed to include elements such as globe preservation, lacrimal gland function, patient satisfaction with visual function, in addition to standard measures of visual acuity. Subjects were treated with vismodegib 150 mg daily for a planned duration of 12 months prior to being offered curative surgery. Of the 31 patients who received vismodegib, whose disease could be assessed with physical examination measurements, 19/31 (56%) had a CR, 10/31 (29%) had a partial response (PR), and 2/31 (6%) had stable disease (SD). Similar response rates were seen on CT/MRI imaging, and no patients progressed while on treatment. Maximum response was seen at 6 months in this trial, and many patients opted for surgery prior to the end of planned 12-month treatment. All patients on trial maintained a VAWS score of >21 points at 12 months or post-operatively, where a score of 21 points was the prespecified benchmark considered a positive clinical outcome, indicating globe preservation plus at least one additional aspect of visual function. When comparing VAWS scores at 12 months or post-operatively to screening scores, 27/33 (82%) had stable or improved VAWS scores, 5/33 (15%) had minor decline, and only 1/33 (3%) had a major decline of 5+ points in their VAWS score. 27 patients in this trial ultimately underwent excision following treatment with vismodegib and 18/27 (67%) had complete histologic response with no residual disease, 6/27 (22%) had residual disease with clear margins, and 3/27 had positive margins (49). A subsequent analysis of residual disease showed proliferation of hedgehog active “micro tumors” - enriched for a W535L mutation in *SMO* (50). These lesions were described as relatively superficial. This underscores the need for resection of residual cutaneous disease post-vismodegib treatment, and the need for continued close surveillance for recurrence. These findings together provided additional evidence for neoadjuvant vismodegib use prior to resection in order to improve visual function and clinical outcomes. One major limitation of this trial is the lack of prolonged follow-up for recurrence which limits assessment of long-term outcomes with a neoadjuvant approach followed by curative surgery.

### Immune checkpoint inhibitors for basal cell carcinoma

Despite the high response rates and success with HHI’s in patients with BCC, most patients ultimately progress or are intolerant of HHI’s. A multi-center phase 2 trial examined the efficacy of anti PD-1 therapy with cemiplimab in patients with locally advanced or metastatic BCC who had progressed or were intolerant of HHI’s, or whose response to HHI was no better than stable disease after 9 months of treatment. This trial enrolled 84 subjects with laBCC and 75/84 (89%) of these patients had BCC of the head and neck who were not candidates for curative surgery or radiotherapy. Subjects received 350 mg of IV cemiplimab every 3 weeks until disease progression or unacceptable toxicity. In the second-line setting, the ORR was around 31%, with 6% CR and 25% PR. There was also 49% SD, for a combined disease control rate of around 80%. Notably, there were a significant number of patients who derived long-lasting benefit from this treatment with durable disease control rate of 60%, defined as no progression for at least 182 days. In terms of safety, there were expected numbers of immune-related adverse effects including colitis, hepatitis and adrenal insufficiency, consistent with those seen with PD-1/PD-L1 inhibitors in other diseases (51). While this trial did not specifically focus on a neoadjuvant approach with immune checkpoint inhibitor prior to any planned curative surgical intervention, it provided another treatment option for patients with locally advanced disease.

### Discussion of systemic therapies for basal cell carcinoma

**Figure 5:**
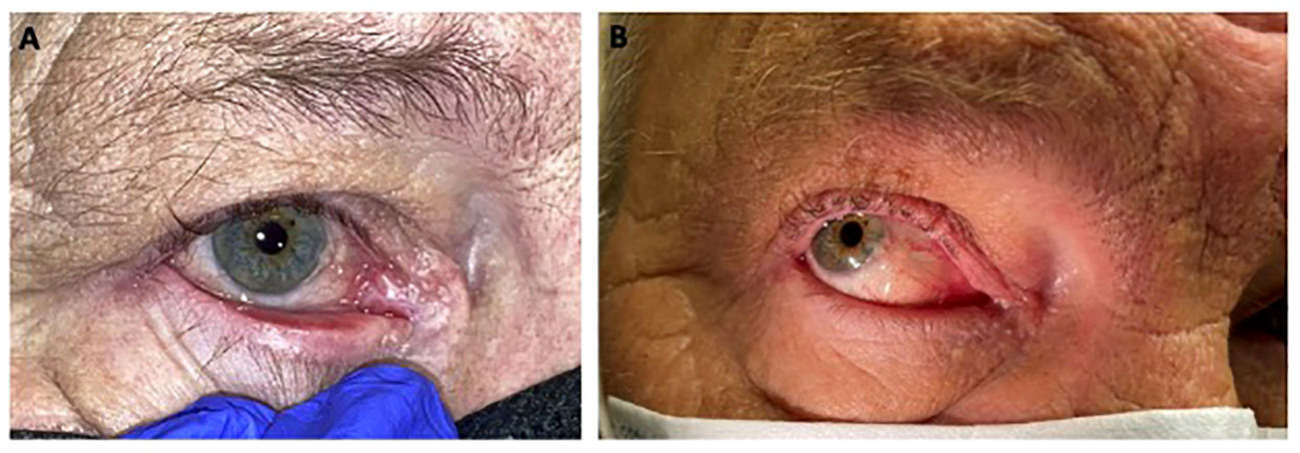
**(A)** 65 year old male with right medial canthal basal cell carcinoma, nodular type. **(B)** Near complete response after 4 weeks of continuous treatment with vismodegib.

The management of BCC in sensitive anatomic locations such as the periocular region remains challenging. Front-line curative surgery may result in significant loss of visual function and disfigurement. While HHI’s and anti-PD-1 inhibitor (cemiplimab) represent effective treatment options for locally advanced and metastatic BCC, their role in the neoadjuvant setting prior to curative surgery remains unclear. The VISMONEO and VISORB trial provide proof of concept that neoadjuvant treatment with HHI’s followed by surgical resection is a reasonable treatment consideration for select patients. Treatment with HHI’s can result in surgical downstaging and can allow for subsequent surgery with preservation of visual structures and functions that would otherwise be impaired by surgery alone. See our case example of a patient with BCC of the medial canthus that was successfully downstaged with pre-operative vismodegib (**Figure 5**). The optimal duration of pre-operative treatment remains unknown. VISMONEO had a median treatment duration of 6 months and VISORB planned for 12 months of treatment, but actual times until resection varied significantly between patients in both studies. Long-term follow-up with this approach remains limited, but the high relapse rates in patients who did not undergo surgery from VISMONEO highlight the ongoing need for surgical resection in this disease. This may be due to more of a cytostatic effect from hedgehog pathway inhibition rather than cytotoxic effect or related to the development of resistance mutations such as SMO W535L throughout the signal transduction pathway.

For patients with laBCC who do not respond to or are intolerant of HHIs, cemiplimab is an appropriate second-line treatment with an ORR of 31% and a high disease control rate of 80% which can be durable. However, there is limited evidence to support use of anti-PD-(L)1 inhibitors prior to surgery and so this should primarily be reserved for patients participating in clinical trials or whom curative surgery is not feasible following HHIs. There is insufficient evidence in the neoadjuvant setting to support the use of cytotoxic chemotherapy for laBCC. Given the complexity of treating patients with BCC in such a sensitive location, multi-disciplinary consultation while carefully considering patient goals and values is essential in tailoring treatment plans to an individual patient. Future prospective studies in this field should endeavor to validate a neoadjuvant approach with HHI therapy, followed by curative surgery with long-term follow-up. There may also be a role for adjuvant treatment with either systemic therapy or radiation for these patients.

## Squamous cell carcinoma of the skin

Squamous cell carcinoma (SCC) of the skin is the second most common skin cancer behind BCC. Around 40% of SCCs are found on the head and neck (52). Similar to BCC, the primary risk factor for SCC is chronic UV exposure, but other important risk factors include immunosuppression, fair skin, advanced age, precancerous skin lesions, and chronic skin infections or inflammatory skin conditions. Most cutaneous SCCs are not invasive and can typically be managed with resection or topical treatments, and only rarely can become invasive or metastatic. Due to the association with chronic UV exposure, SCC also bears a relatively high tumor mutational burden with a median of 45.2 mutations/Mb (40). SCC becomes the most common skin cancer in immunocompromised individuals, such as those after solid organ transplantation (53, 54). The discovery of high TMB and interaction of the immune system with SCC have led to the logical conclusion that immune checkpoint inhibitors may represent a promising treatment option for patients with SCC not amenable to curative surgery.

### Chemotherapy for squamous cell carcinoma of the skin

Much of the data regarding cytotoxic chemotherapy for cutaneous SCC in the periocular region is extrapolated from studies of primary head and neck SCC. Platinum-based regimens such as cisplatin with 5-fluorouracil or carboplatin with paclitaxel, have primarily been used (43). Chemotherapy and/or EGFR inhibitors, like cetuximab, can be given with or without radiotherapy (55–57). There is limited data on the efficacy of chemotherapy or EGFR inhibitors in the neoadjuvant setting. Chemotherapy is now generally reserved for patients who cannot safely receive immune checkpoint inhibitors, such as solid organ transplant recipients or individuals where the risk of severe immune-related adverse events outweigh the anti-tumor benefits.

### Immune checkpoint inhibitors for squamous cell carcinoma of the skin

Cemiplimab, an anti-PD-1 monoclonal antibody, was the first immune checkpoint inhibitor approved for cutaneous SCC. Pooled data of phase 2 trials looking at 193 patients with locally advanced (laSCC) or metastatic (mSCC) cutaneous SCC, most originated in the head and neck region, showed an ORR of 46.1%. This included 16.1% CR and 30.1% PR. Two dosing schedules were used which may have affected results (58, 59). In one of the phase 2 trials, the reason surgery was not feasible was included. Among patients evaluated for surgical candidacy, 26% had substantial local invasion that precluded complete resection, 38% had lesions in an anatomically challenging location for which surgery might result in severe disfigurement or dysfunction, 32% in the same location of prior surgery and additional curative resection was deemed unlikely.

Pembrolizumab, another anti-PD-1 monoclonal antibody, was studied in a similar capacity. The phase 2 CARSKIN trial evaluated first-line pembrolizumab for patients with locally advanced or metastatic SCC and reported an ORR of 42% across all patients with 7% CR. 32% of patients had progressive disease on treatment. Two patients from this trial were noted to have initially unresectable regional disease who eventually underwent conservative surgery after initial treatment with pembrolizumab (60). Long-term outcomes of these patients were not reported. KEYNOTE-629 examined pembrolizumab for locally advanced, recurrent, and metastatic SCC not amenable to curative surgery or radiation. This trial reported an ORR of 50% for laSCC with 16.7% complete responders (61).

The relatively high response rates, with some with some complete responders, to anti-PD-1 therapy in patients with locally advanced cutaneous SCC have prompted investigation into the neoadjuvant approach. A phase 2 study evaluated cemiplimab in 79 patients with resectable stage II-IV cutaneous SCC. 91% of the subjects had their primary tumor site on the head and neck (62). Patients received up to four doses of cemiplimab prior to surgery with curative intent. Of those who underwent surgery, a pathologic CR was seen in 51% of patients per independent review, and an additional 13% had a major pathologic response defined as <10% viable tumor cells remaining. At the time of data cutoff, none of the patients who underwent curative surgery had disease recurrence. Eligibility included patients where orbital exenteration would be required. The authors reported at least two patients who had a pathologic complete response who were spared orbital exenteration as a result of response to cemiplimab. Eight patients had disease progression prior to receiving the planned four doses of cemiplimab and nine patients did not undergo surgery in the protocol specified study window. 3/9 patients progressed on cemiplimab.

The optimal number of neoadjuvant anti-PD-1 doses remains uncertain for locally advanced cutaneous SCC. Furthermore, the role of adjuvant anti-PD-1 following surgery is unclear. The NEO-CESQ study is a phase II study seeks to evaluate the efficacy of neoadjuvant plus adjuvant cemiplimab in patients with surgically resectable, high-risk stage III/IV (M0) cutaneous SCC. In this study, 2 cycles of cemiplimab are administered in the neoadjuvant setting, followed by adjuvant cemiplimab for one year after surgery. 23 subjects were enrolled in the initial data report. Pathologic CR was seen in 9/23 (39%) patients and near pathologic CR in 2/23 (8%) patients (63). Longitudinal follow up, including relapse free survival, is still ongoing and a larger cohort study is anticipated.

Several small case series have since been published describing successful use of anti-PD-1 therapy for SCC in the periocular region. A study of 5 patients with SCC who were recommended orbital exenteration, but refused, were treated with anti-PD-1 therapy. Four patients had a CR without need for surgery, and one progressed despite treatment (64). Another series of 7 patients whose lesions were either rapidly progressive or not amenable to additional surgery/radiation were treated with anti-PD-1 therapies and all patients responded. Five of these patients went on to surgery after achieving a clinical response and the other two who had extensive prior surgery and radiation achieved a complete response without further treatment (65). A series of 11 patients with orbital SCC that would require exenteration were treated with cemiplimab and 9/11 (82%) had a CR without need for surgery, with only one patient progressing on cemiplimab and another patient entering hospice for a second cancer (66). The largest such study of 13 patients in Israel of periocular SCC with orbital invasion treated with cemiplimab showed an overall response in 9/13 (69.2%) of patients with 7/13 (53.8%) achieving a CR. No patients required orbital exenteration, but two had progressive disease and one patient died of treatment related myocarditis (67).

### Discussion of systemic therapies for squamous cell carcinoma of the skin

These studies have several key conclusions and limitations when considering the approach to managing cutaneous SCC in the periocular region. They all enrolled a high portion of cutaneous SCC located on the head and neck, but further specificity or proximity to the orbit is not explicitly stated. The bulk of these trials focused on patients with unresectable disease that was either locally advanced or metastatic. At least two phase 2 studies have evaluated a neoadjuvant approach with cemiplimab, which primarily enrolled subjects with resectable disease. Patients who successfully went on to surgery achieved pathologic response rates in nearly half the enrolled subjects. In one of two studies, those who underwent resection have not had disease recurrence to date with limited follow-up, but this fact underscores the need for surgical management. While the authors of these studies point to several cases where surgery became feasible or orbital exenteration was spared, the primary outcomes of these trials were response rates and safety events. This contrasts with VISMONEO and VISORB studies in BCC which specifically enrolled patients with unresectable disease or resectable with major morbidity or functional aesthetic, had primary outcomes focused on surgical staging and preservation of visual structures/function. Additionally, several patients treated with neoadjuvant cemiplimab for cutaneous SCC had resectable disease at baseline and progressed to unresectable disease, representing a significant loss of potential for curative treatment.

With these limitations in mind, it remains unclear whether a neoadjuvant approach with immunotherapy represents the optimal management of periocular SCC. Many would argue that there remains insufficient evidence to support this approach, especially in patients with disease that can still be managed with curative resection. However, given the high response rate seen with immune checkpoint inhibitors and some reports of successful use in periocular SCC, it represents a possible treatment approach in a highly selected patient population for whom there are not more definitive treatment options.

In summary, the current standard of care remains definitive surgical resection, but neoadjuvant immune checkpoint inhibitor should be considered in patients who may be borderline candidates for surgery or decline surgery due to functional or aesthetic risk, consistent with NCCN guidelines. Patients undergoing this approach should be counseled that progression of disease while on treatment is possible and may threaten their ability to undergo curative surgery in the future. Additional studies are needed to directly address the question of whether unresectable tumors in the periocular region can be successfully made resectable and potentially cured with neoadjuvant systemic therapies. There also remains no standard adjuvant treatment approach after surgical resection, though studies looking at adjuvant anti-PD-1 is ongoing. Based on data from other malignant conditions, there may be a role for adjuvant radiation in cutaneous SCC.

## Cutaneous melanoma

Melanoma represents only 1% of skin cancers but causes over 80% of skin cancer deaths (68). Melanoma is disproportionately found in the head and neck area, and may affect the skin of the face and eyelid (69). Melanoma arising from the conjunctiva is a rare subtype of melanoma, but its histological behavior appears similar to cutaneous melanoma (70). Mucosal and uveal melanomas are typically more aggressive than cutaneous melanoma, but will not be discussed in this review. The main risk factor for cutaneous melanoma is UV exposure, however, the development of melanoma is more closely tied to sunburns and other types of intermittent sun exposure (71). This is in contrast to chronic UV exposure which is more strongly linked to the development of BCC and SCC. Other risk factors for melanoma include fair skin, indoor tanning, high number of moles, family history, inherited cancer syndrome (germline mutations in *CDKN2A*, *CDK4*, *P53*, *MITF*), increasing age, and immunosuppression.

Melanoma has been identified as a highly immunogenic tumor which may respond favorably to immunotherapy. Initial therapeutic options for melanoma included efforts at modulating the immune system with the use of cytokines, interleukins, peptide-based vaccination, and eventually immune checkpoint inhibitors. In terms of targeted therapies, roughly 40-60% of cutaneous melanoma possess a *BRAF* V600 mutation, which can be effectively treated with inhibitors of BRAF and the downstream target MEK (72). Prior to 2011, when the first immune checkpoint inhibitors and BRAF targeting medications were approved by the United States Food and Drug Administration (FDA), data from the Surveillance, Epidemiology, and End Results (SEER) program showed a 5-year survival of 16.1% for patients with distant metastases from cutaneous melanoma. Since then, several immune checkpoint agents targeting CTLA-4, PD-1, LAG-3, and targeted inhibitors of BRAF and MEK have been approved either alone or in combination. Updated data from 2019 SEER program now show the 5-year survival percentage has more than doubled at 35.1% with the use of these agents (73).

### Chemotherapy for cutaneous melanoma

A variety of cytotoxic chemotherapies have been tried in metastatic melanoma prior to the development of immune-modulating therapies. All chemotherapeutic agents had disappointing response rates and no single agent or combination has ever been shown to increase overall survival. Dacarbazine and temozolomide have been the most commonly used agents, with response rates reported around 10-20% and PFS around 2 months (74, 75). More aggressive combination chemotherapy regimens such as the Darthmouth regimen of dacarbazine, cisplatin, carmustine and tamoxifen reported a similar response rate of 18.5% and did not increase survival (76). There is little to no data on efficacy of chemotherapy in the neoadjuvant setting or specifically in the periocular region.

### Immune checkpoint inhibitors for cutaneous melanoma

The pivotal CheckMate 067 trial established the preferential use of combination ipilimumab 3 mg/kg with nivolumab 1 mg/kg in patients with unresectable or metastatic melanoma. This regimen demonstrated high response rates, durable responses, and improved overall survival compared to ipilimumab monotherapy (median overall survival of 72.1 vs 19.9 months), albeit with relatively high toxicity rates (77). To counteract the high toxicity rates, CheckMate 511 demonstrated significantly lower incidence of high-grade toxicities with the flipped dosing of ipilimumab 1 mg/kg with nivolumab 3 mg/kg (78). The newest immune checkpoint target, relatlimab targeting LAG-3, was recently studied in combination with nivolumab as an alternative combination ICI regimen to ipilimumab/nivolumab for patients with unresectable or metastatic disease (79). Several studies showed the efficacy of adjuvant immune checkpoint inhibition (anti-CTLA-4 and anti-PD-1) in reducing relapse free survival in resected melanoma at high risk of recurrence (80, 81).

The success of immune checkpoint inhibitors in the metastatic and adjuvant setting has led to the possibility of neoadjuvant treatment prior to resection. Immunotherapy administration prior to resection may result in enhanced exposure of tumor antigens to a broader range of immune effector cells and more robust T cell response, thus leading to improved overall antitumor response (82). This approach is highly relevant for melanoma arising in the periocular region where definitive resection with appropriately wide surgical margins may not be feasible. Melanoma in this area can often present without nodal involvement or distant metastatic disease which would otherwise warrant systemic treatment. However, neoadjuvant treatment has been approached with extreme caution even in patients with highly morbid but resectable disease due to concern that any delay in surgery or lack of response might allow this disease to progress or metastasize.

As of 2023, neoadjuvant trials of immune checkpoint inhibitors in locally advanced, resectable melanoma have mainly focused on patients with stage IIIB-IV. This includes primarily patients with clinically detectable lymph node disease with a small proportion of subjects with resectable oligometastasis. In this population, risk of recurrence is high and trials have mainly focused on outcomes such as recurrence free survival. One of the first exploratory neoadjuvant studies compared single-agent nivolumab 3 mg/kg for up to 4 doses vs combination ipilimumab 3 mg/kg with nivolumab 1 mg/kg for up to 3 doses in patients with resectable stage IIIB-IV melanoma (83). Response rate by RECIST version 1.1 in the nivolumab group was low at 25%, compared to 73% in the combination ipilimumab/nivolumab group. This trial had to be stopped early by the data safety monitoring board based on significant disease progression in the nivolumab monotherapy group which prevented surgical resection. Additionally, toxicity in the combination arm was high and contributed to trial cessation, with 73% of patients in the group experiencing grade 3 or higher treatment-related adverse events.

After this exploratory trial, the phase 1b OpACIN trial randomized 20 patients with resectable stage III melanoma to either 2 doses of ipilimumab 3 mg/kg with nivolumab 1 mg/kg prior to surgery and 2 doses after surgery, or all 4 doses given after surgery (84). Patients administered 2 doses of ipilimumab/nivolumab had a pathologic CR rate of 33% but high overall pathologic response rate of 78%. Importantly, at long term follow-up, pathologic CR was a reliable surrogate for relapse-free survival and overall survival. Based on these results, the phase 2 OpACIN-neo trial sought to optimize the dosing and schedule of combination ipilimumab/nivolumab prior to resection and enrolled a total of 86 patients into 3 arms (85). The optimal group from this trial (n=30) received 2 doses of ipilimumab 1 mg/kg with nivolumab 3 mg/kg prior to surgery had 57% pathologic CR, and 77% pathologic response rate overall. By imaging, 57% achieved an objective response. Only two patients in this arm experienced disease progression prior to planned surgery, one with local progression and one who developed distant metastasis. The expansion cohort of 99 patients from OpACIN (published as the PRADO trial) used this optimized neoadjuvant schedule to determine if major pathologic response to neoadjuvant ipilimumab/nivolumab could safely spare total lymph node dissection. Similar rates of pathologic CR (49%) and pathologic response rate (72%) were observed. In this trial, 13% of patients experienced progression, 6% of whom had distant metastases prior to surgery. Three patients had surgery delayed and one patient could not undergo any surgery due to treatment related adverse events (85, 86). Published in 2023, the SWOG 1801 trial compared pembrolizumab given before and after surgery vs adjuvant pembrolizumab alone in patients with resectable stage IIIB-IV melanoma. The neoadjuvant-adjuvant group in this study reported a pathologic CR of 21%. Of 144 patients in the neoadjuvant group, 12 patients had had disease progression and could not go on to resection and one had treatment related adverse effects that precluded surgery. Several patients were also unable to proceed with adjuvant therapy after surgery due to neoadjuvant treatment related toxicity. Event free survival at 2 years was 72% in the neoadjuvant-adjuvant group compared to 49% in the adjuvant only group, establishing the practice-changing benefit of neoadjuvant anti-PD-1 therapy in this population (87).

### BRAF/MEK inhibitors for cutaneous melanoma

Several combinations of BRAF/MEK inhibitors have been approved in locally advanced or metastatic melanoma including dabrafenib/trametinib, encorafenib/binimetinib, and vemurafenib/cobimetinib. These agents have demonstrated even higher overall response rates (64-70%) and more rapid responses compared to immune checkpoint inhibitors, but with shorter duration of responses. Median duration of response with combined ipilimumab/nivolumab was not reached but had a lower 95% CI of 61.9 months, compared to only 12.0 months with dabrafenib/trametinib, 18.6 months with encorafenib/binimetinib, and 14.7 months with vemurafenib/cobimetinib (88–90). Dabrafenib/trametinib has also been approved for use in the adjuvant setting adjuvant to reduce risk of recurrence following resection of locally advanced and/or node-positive disease (91).

The high response rate and rapid responses sparked interest in neoadjuvant BRAF/MEK inhibition in resectable melanoma. The phase 2 NeoCombi trial enrolled 35 patients with *BRAF* V600-mutated melanoma with clinically detectable lymph nodes to receive combination BRAF/MEK inhibition with dabrafenib/trametinib for 12 weeks prior to surgery. This trial demonstrated a high response rate, with 100% patients having a pathologic response, and 49% of patients having a pathologic CR. No patients progressed while on neoadjuvant dabrafenib/trametinib. An exploratory endpoint of ease of surgical resection was reported as improved after 12 weeks of neoadjuvant treatment in 46% of patients and unchanged in 54% of patients. Despite the high response rate seen, 57% of patients in this trial had recurrence at time of data cutoff (92). The high recurrence rate was surprising as it differed significantly from immunotherapy-based studies which had lower recurrence rates, especially in those who achieved a pathologic complete response. The NeoTrio is the first trial studying the combination of anti-PD-1 therapy with concurrent BRAF/MEK inhibition compared with either approach alone (93). In the 20 patients treated with pembrolizumab plus dabrafenib/trametinib, preliminary results showed higher pathologic CR rate than either approach alone, but with more toxicity. As of 2023, the longitudinal results are not available.

### Discussion of systemic therapies for cutaneous melanoma

The above trials have provided proof of concept that neoadjuvant immunotherapy and/or BRAF/MEK inhibition can lead to clinical and pathologic response before surgery and potentially improve long term outcomes for patients with resectable melanoma. However, there has been little research focused on melanoma occurring in the periocular region. Given its diverse anatomic potential, melanoma is less likely to occur on the head/neck compared to other cutaneous malignancies. The percentage of patients with head and neck melanomas accrued on trials is generally lower compared to trials examining SCC or BCC. The primary endpoints of neoadjuvant trials in melanoma offer insight into long-term relapse rate, but do not examine the resectability of tumors or the impact of resection on local structures, a crucial component of managing periocular disease. Neoadjuvant immunotherapy also confers significant risk in terms of toxicity and chance of progression in the pre-operative setting. Grade 3 or higher toxicity rates ranged from 12% to 50% depending on dosing and schedule, and in many cases rendered patients unfit for surgery. Additionally, there is a clinically significant risk of disease progression even with combination immunotherapy. The risk of progression was lower in patients treated with neoadjuvant dabrafenib/trametinib in the NeoCombi study, but this treatment approach also seemed to confer less long-term benefit and higher risk of recurrence. Taken together, neoadjuvant immunotherapy is a promising tool for improving long-term relapse free survival, but its effect on surgical resection and outcomes remains unknown. BRAF/MEK inhibition has high response rates and may improve ease of surgery but may be inferior at preventing future relapse given their historic limited duration of response in the unresectable/metastatic setting. There is insufficient evidence to suggest unresectable periocular melanoma may become resectable or that long term outcomes would be improved beyond what is reported with current neoadjuvant approaches. Additional studies combining less toxic combinations of immunotherapy such as PD-1 and LAG-3 inhibition are underway, as well as increasingly aggressive approaches of immunotherapy with BRAF/MEK inhibition seen in the NeoTrio trial. These studies, as well as studies focusing on melanoma in anatomically sensitive areas will be necessary before considering any deviation to the current standard of care management of cutaneous melanoma.

## Merkel cell carcinoma

Merkel cell carcinoma (MCC) is a rare form of skin cancer with unique neuroendocrine features. It is an aggressive form of skin cancer which behaves similarly to other high-grade neuroendocrine carcinomas, with early metastasis and a high case-fatality rate. Metastatic disease has a poor prognosis, with 5-year survival rates around 13.5% (94). Tumorigenesis of MCC is typically attributed to either chronic UV exposure with high mutational burden or the presence of clonally integrated Merkel Cell polyomavirus. Patients with compromised immune systems and the elderly are at higher risk of developing virally-driven MCC.

Management of limited stage MCC consists of definitive wide local excision of the primary tumor followed by adjuvant radiation therapy, based on presence of adverse risk factors. Sentinel lymph node mapping/biopsy is generally performed at the time of the excision. Clinically detectable lymph node disease often requires complete lymph node dissection and adjuvant radiotherapy to the LN basin is often recommended for node-positive disease.

### Chemotherapy for Merkel cell carcinoma

Chemotherapy with platinum-based regimens were historically used as standard of care for unresectable or metastatic MCC. Regimens consisting of a platinum-based agent (cisplatin or carboplatin) with etoposide or triplet therapy with cyclophosphamide, doxorubicin and vincristine (CAV) demonstrated high response rates between 60-75% but responses are often short lived (95). Most patients progress within 2-4 months of cytotoxic chemotherapy. Toxicity was relatively high and survival benefit of these treatments in advanced disease remains controversial. Isolated reports describe the successful use of neoadjuvant cisplatin, etoposide and cyclophosphamide prior to surgery in two patients, one of whom had MCC on the cheek (96).

### Immune checkpoint inhibitors for Merkel cell carcinoma

As of 2023, the current standard of care for treatment of recurrent, unresectable, or metastatic disease is immune checkpoint inhibitors with FDA-approved agents such as avelumab (anti-PD-L1), pembrolizumab (anti-PD-1), and retifanlimab (anti-PD-1). These agents result in slightly lower response rates compared to chemotherapy, between 32-64%, but with greater durability of response (97). Small cohort studies of systemic-treatment naïve advanced MCC patients treated with combination ipilimumab with nivolumab have demonstrated response rates of 58-100%, but ongoing larger prospective studies are currently in progress (98). Recent phase 2 studies have also shown evidence for ipilimumab with nivolumab in combination with radiation which resulted in response rates between 52-72% for patients with advanced disease (99). There is no current recommendation for adjuvant systemic therapy, but adjuvant anti-PD-(L)1 is currently being investigated in clinical trials (NCT03712605, NCT03271372, NCT02196961, NCT04291885).

### Discussion of systemic therapies for Merkel cell carcinoma

Neoadjuvant systemic therapy has been considered when MCC is present in highly sensitive areas of the body, such as the eyelid. In these situations, platinum-based chemotherapy regimens have been used due to high response rates, but evidence for this approach consists primarily of case reports/series (96). The phase 2 CheckMate 358 trial examined the role of neoadjuvant nivolumab in 39 patients with resectable MCC. Patients received 2 doses of nivolumab prior to planned surgery. Two patients could not receive surgery due to treatment related adverse events, and one patient due to tumor progression. Of the patients who received surgery, 46.2% achieved a pathologic CR, and 61.5% achieved either a complete or major pathologic response. By imaging, 87.9% of patients showed any radiographic tumor reduction and 54.5% of patients had reduction in >30% (100).

For patients with periocular MCC, definitive resection or radiation may not be feasible and a trial of either anti-PD-(L)1 or chemotherapy should be considered before definitive surgery. Chemotherapy has high response rates and may be used for very symptomatic periocular tumors, but not every patient is an appropriate candidate for cytotoxic chemotherapy. The optimal treatment duration and timing of surgery may be challenging to coordinate given the limited response duration and unclear recurrence rate following resection. Due to this uncertainty, we suggest avoiding preoperative chemotherapy unless a rapid response is needed or if a patient is not eligible for immune checkpoint inhibitors. Anti-PD-1 therapy with nivolumab has demonstrated tumor shrinkage prior to surgery with relatively high pathologic response rates. With this approach, some patients may progress or have toxicities which preclude surgery. As of 2023, the role of adjuvant immune checkpoint inhibitor is uncertain, therefore post-surgical outcomes in the periocular region remain unknown. If a pre-operative approach is warranted, anti-PD-(L)1 therapy is generally preferred over chemotherapy. There are also several promising treatments currently under investigation in locally advanced or metastatic MCC. These include somatostatin receptor-based therapies and somatostatin analogs, pazopanib and other targeted therapies, oncolytic viral therapy with talimogene laherparepvec, or combination immunotherapy. In the future, a multi-target approach may lead to improved response rates and outcomes for neoadjuvant therapy in MCC.

### Sebaceous gland carcinoma

Sebaceous gland carcinoma (SGC) is a rare skin cancer that has high incidence in the periocular region, with around 70% of all cases occurring on the head and neck and up to 40% of cases arising on the eyelid (101). Diagnosis can be challenging as lesions can easily be mistaken for other benign eyelid lesions, such as chalazion. Risk factors are similar to other skin cancers and include immunosuppression, advanced age, and UV exposure. SGC can also be seen in younger patients without these risk factors in Muir-Torre syndrome, a type of hereditary non-polyposis colorectal cancer (HNPCC) syndrome characterized by mutations in DNA mismatch repair enzymes (102). Localized lesions are typically managed with either Mohs surgery or wide local excision and radiation if surgery is not feasible. Adjuvant radiotherapy is considered in high-risk situations such as positive surgical margins, perineural invasion, or in cases with lymph node metastases. There is currently no standard systemic therapy recommended for cases of recurrent or metastatic disease. There are case reports describing the use of platinum, anthracycline or taxane-based chemotherapy regimens and immune checkpoint inhibitors. The anti-PD-1 inhibitor pembrolizumab has been used alone or in combination with chemotherapy in microsatellite instability high and low metastatic SGC (103–105). There is limited data to support neoadjuvant systemic therapy for periocular SGC. One retrospective case series of 8 patients receiving neoadjuvant platinum-based chemotherapy reported an ORR of 87.5% with 71% reduction in tumor diameter, with 2 patients experiencing a radiographic complete response. When compared to a cohort of patients who did not receive neoadjuvant chemotherapy, patients treated with chemotherapy were more likely to have eyelid-sparing surgery (106). There is one case report describing use of neoadjuvant pembrolizumab in a patient with Muir-Torre syndrome. The patient received 2 doses of pembrolizumab which resulted in tumor shrinkage to less than 1/3 its original size prior to Mohs surgery (107). While data is extremely limited on the use of neoadjuvant systemic therapy for periocular SGC, it is reasonable to consider either chemotherapy or immune checkpoint inhibitors prior to surgery. In cases of Muir-Torre syndrome with mismatch repair deficiency or microsatellite instability-high tumors, there is evidence in other tumor types with resistance to chemotherapy, to preferentially use immune checkpoint inhibitors (108).

## Future directions

As of 2023, surgical, reconstructive and radiotherapeutic techniques for skin cancers have been relatively stable over time, but systemic therapies for skin cancers are evolving rapidly. Several areas of research are needed to better elucidate the optimal management of periocular skin cancers. First, larger phase 3 trials are necessary to confirm expected responses in each tumor type. Second, dedicated neoadjuvant studies with eligibility criteria that includes commonly presented periocular malignancies will need to be performed, many of which are underway (**Table 4**). These will need to be performed in resectable cancers to see if the combination of immunotherapy followed by surgery leads to improved overall outcomes or if surgery can be avoided entirely. Studies should also be performed in patients with unresectable disease to determine whether or not these therapies can make tumors amenable to curative resection. Ideally, these trials would be focused on tumors in the periocular area to clarify treatment approaches in this sensitive anatomic area. The rarity of some tumor types in the periocular region may limit the ability to conduct large scale trials.

**Table 4:** Active clinical trials of systemic treatment for periocular malignancies.

ClinicalTrials.gov ID	Disease of Interest	Disease Stage	Treatment
**NCT04154943**	Cutaneous SCC	Resectable stage II-IV	Neoadjuvant cemiplimab q3w for up to 4c
**NCT04710498**	Cutaneous SCC	Resectable locally advanced	Neoadjuvant atezolizumab q3w for 3c
**NCT04808999**	Cutaneous SCC	Resectable stage II-IV	Neoadjuvant pembrolizumab q3w for 2c, then adjuvant pembrolizumab for 15c
**NCT05025813**	Cutaneous SCC	Resectable stage II-IV	Neoadjuvant pembrolizumab q3w for 4c, then surgery +/- EBRT, then adjuvant pembrolizumab for 17c
**NCT04315701**	Cutaneous SCC	Resectable stage I-III	Neoadjuvant cemiplimab q3w for up to 3c
**NCT04620200**	Cutaneous SCC	Resectable stage II-IVa	Neoadjuvant nivolumab +/- ipilimumab q2w for 2c
**NCT04428671**	Cutaneous SCC	Resectable high-risk disease	Neoadjuvant cemiplimab q3w for up to 3c, then adjuvant cemiplimab q3w for up to 18c
**NCT04975152**	Cutaneous SCC	Resectable stage II-IV	Neoadjuvant cemiplimab q3w for up to 9c, then adjuvant cemiplimab q3w for up to 8c
**NCT02324608**	Cutaneous SCC	Resectable high-risk disease	Neoadjuvant cetuximab weekly for 8w
**NCT03035188**	Basal cell carcinoma	Resectable large (>2 cm) disease	Neoadjuvant vismodegib daily for up to 12w
**NCT04323202**	Basal cell carcinoma	Resectable large (>2 cm) disease	Neoadjuvant pembrolizumab q3w for 4c, then adjuvant pembrolizumab for 13c
**NCT03534947**	Basal cell carcinoma	Resectable cosmetically challenging disease	Neoadjuvant sonidegib for 12w, then imiquimod for 6w or surgery or best supportive care
**NCT05496036**	Merkel cell carcinoma	Resectable stage I-III	Neoadjuvant pembrolizumab 400 mg for 1c, then adjuvant pembrolizumab for 1 year
**NCT04869137**	Merkel cell carcinoma	Resectable stage II-IV	Neoadjuvant pembrolizumab q3w for 2c + lenvatinib, then surgery +/- adjuvant radiotherapy, then adjuvant pembrolizumab for 17c
**NCT04975152**	Merkel cell carcinoma	Resectable stage I-II	Neoadjuvant cemiplimab q3w for 9c, then adjuvant cemiplimab for up to 8c
**NCT04020809**	Melanoma	Resectable stage I-II	Neoadjuvant atezolizumab q3w for 2c

SCC, squamous cell carcinoma; q, every; w, week; c, cycle; EBRT, external beam radiotherapy.

## Conclusion

Optimal management of periocular skin cancers depends on tumor type, size, and involvement of orbital and adnexal structures. Many patients will not be able to undergo curative surgery or radiation without severe disruption of visual structures and facial appearance. Neoadjuvant treatment with chemotherapy, targeted therapies, and immune checkpoint inhibitors should be considered depending on the tumor type, tumor molecular profiling, expected response rates, and candidacy for systemic treatment. Several neoadjuvant studies are under active investigation which may allow accrual of locally advanced periocular malignancies without nodal involvement.

## Author contributions

DT: Conceptualization, Investigation, Writing – original draft, Writing – review & editing, Resources, Visualization. LH: Investigation, Resources, Visualization, Writing – original draft, Writing – review & editing. SL: Conceptualization, Investigation, Resources, Supervision, Validation, Visualization, Writing – original draft, Writing – review & editing, Funding acquisition. AB: Investigation, Resources, Supervision, Validation, Visualization, Writing – original draft, Writing – review & editing, Conceptualization. VM: Conceptualization, Investigation, Resources, Supervision, Validation, Visualization, Writing – original draft, Writing – review & editing.
